# *Cordyceps militaris* and *Armillaria mellea* formula alleviates depressive behaviors *via* microglia regulation in an unpredictable chronic mild stress animal model

**DOI:** 10.1016/j.jtcme.2024.05.003

**Published:** 2024-05-16

**Authors:** Yu-En Lin, Hui-Ping Lin, Kuan-Hung Lu, Yun-Ju Huang, Suraphan Panyod, Wei-Ting Liu, Yun-Sheng Lu, Mei-Hsing Chen, Lee-Yan Sheen

**Affiliations:** a Institute of Food Science and Technology, College of Bioresources and Agriculture, National Taiwan University, Taipei, Taiwan; b Institute of Food Safety and Health, National Taiwan University, Taipei, Taiwan; c Institute of Environmental and Occupational Health Sciences, National Taiwan University, Taipei, Taiwan; d Department of Biotechnology and Food Technology, Southern Taiwan University of Science and Technology, Tainan, Taiwan; e Taiwan Agricultural Research Institute, Council of Agricultural, Taichung, Taiwan; f Center for Food and Biomolecules, National Taiwan University, Taipei, Taiwan; g National Center for Food Safety Education and Research, National Taiwan University, Taipei, Taiwan

**Keywords:** *Cordyceps militaris*, *Armillaria mellea*, Depression, Unpredictable chronic mild stress, Microglia

## Abstract

**Background and aim:**

*Cordyceps militaris* (CM) and *Armillaria mellea* (AM) are medicinal mushrooms with potential applications in the treatment of mood disorders, including depression and anxiety. While research suggests that both CM and AM possess anti-inflammatory properties and hold potential for treating depression when administered separately, there is limited knowledge about their efficacy when combined in a formula, as well as the underlying mechanism involving the modulation of microglia.

**Experimental procedure:**

Rats received oral administrations of the low-dose formulation, medium-dose formulation, and high-dose formulation over 28 consecutive days as part of the UCMS protocols. The concentrations of serotonin, dopamine, and the corresponding metabolites in the rat prefrontal cortex and hippocampus were assessed. Blood samples were collected to examine corticosterone levels, and the brains were dissected for evaluating activated microglia morphologies and associated pro- and anti-inflammatory signaling pathways.

**Results and conclusion:**

The CM-AM formula effectively averted abnormal behaviors triggered by UCMS, such as anhedonia and hypoactivity, and decreased the turnover rate of monoamines in both the prefrontal cortex and hippocampus. The formula mitigated the increase in serum corticosterone levels induced by chronic stress. Furthermore, the formula alleviated stress-induced microglia activation in the hippocampus, achieving this by down-regulating hyperactivated pro-inflammatory proteins and up-regulating hypoactivated anti-inflammatory proteins in the hippocampus. The antidepressant-like effects potentially stemming from the regulation of neurotransmitters and immunomodulation, likely by restoring the balance of M1 and M2 microglia fractions in the hippocampus. Consequently, the CM-AM formula could be explored as a prospective complementary and alternative therapy for depression.

## Highlights

• The CM-AM formula alleviated UCMS-induced depression-like behaviors.
• The CM-AM formula normalized monoamine turnover in UCMS-exposed rats.
• The CM-AM formula regulated inflammatory responses in the rat hippocampus.
• The CM-AM formula balanced M1/M2 microglia in the rat hippocampus.

## Graphical abstract

**Figure:**
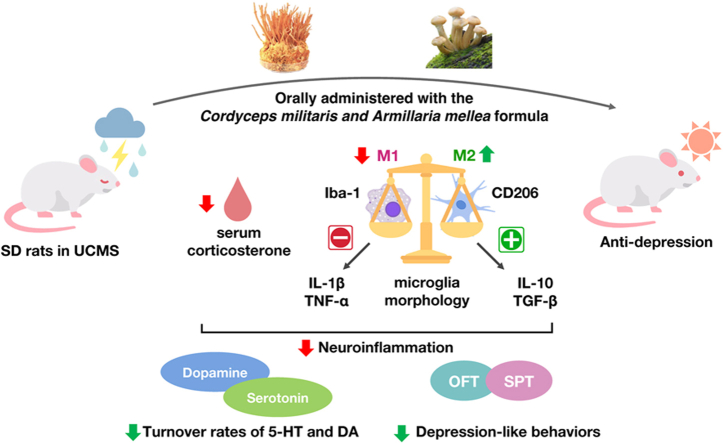

## Abbreviations

alanine aminotransferase: ALT
aspartate aminotransferase: AST
analysis of variance: ANOVA
*Armillaria mellea* (Vahl) P. Kumm: AM
body weight: bw
brain-derived neurotrophic factor: BDNF
carboxymethylcellulose: CMC
central nervous system: CNS
control group: CTL
*Cordyceps militaris* (Linn.) Link: CM
dopamine: DA
electrochemical detector: ECD
enzyme-linked immunosorbent assay: ELISA
ethylenediaminetetraacetic acid: EDTA
fractal dimension: D_B_
high performance liquid chromatography: HPLC
high-dose formulation group: HD
hypothalamic-pituitary-adrenal: HPA
immunohistochemistry: IHC
Institutional Animal Care and Use Committee: IACUC
insulin-like growth factor-1: IGF-1
interleukin: IL
ionized calcium binding adaptor molecule 1: Iba-1
low-dose formulation group: LD
liquid chromatograph/mass spectrometer/mass spectrometer: LC/MS/MS
lipopolysaccharide: LPS
Major Depressive Disorder: MDD
mannose receptor cluster of differentiation 206: CD206
medium-dose formulation group: MD
negative control group: NCTL
no-observed-adverse-effect level: NOAEL
noradrenaline: NA
no significance: ns
nuclear factor kappa B: NF-κB
open field test: OFT
phosphate buffered saline: PBS
positive control group: PCTL
serotonin: 5-HT
sodium dodecyl sulfate polyacrylamide gel electrophoresis: SDS-PAGE
sucrose preference test: SPT
Taiwan Agricultural Research Institute: TARI
traditional Chinese medicine: TCM
transforming growth factor: TGF
tumor necrosis factor: TNF
unpredictable chronic mild stress: UCMS
World Health Organization: WHO
1-octanesulfonic acid sodium salt: SOS
3,4-dihydroxyphenylacetic acid: DOPAC
5-hydroxyindoleacetic acid: 5-HIAA

## 1 Introduction

Major Depressive Disorder (MDD) is a complex mental health condition with profound effects on millions of people worldwide. The symptoms include low mood, reduced interest in once-enjoyable activities, fatigue, cognitive challenges, appetite, and sleep disruptions, and sometimes suicidal thoughts.^1^ However, due to the unpleasant side effects of antidepressants, MDD patients often have low treatment adherence,^2^ making MDD a significant public health concern with social and economic implications.^3^ Exploring alternative approaches like dietary therapies or herbal medicine to prevent or alleviate depression symptoms may help mitigate its progression.

While the imbalance of monoamine neurotransmitters like serotonin (5-HT), dopamine (DA), and noradrenaline (NA) is a known factor in depression,^4^ its pathogenesis is also closely linked to chronic inflammation and dysregulation of the hypothalamic-pituitary-adrenal (HPA) axis.^5^ Normally, glucocorticoids suppress the immune system, inhibiting the secretion of proinflammatory cytokines such as tumor necrosis factor (TNF)-α, interleukin (IL)-1β, and IL-6. However, prolonged stress disrupts this balance, leading to heightened inflammation that can cause neuronal injury or death, increasing the risk of physical and mental illnesses.^6^ Pro-inflammatory cytokines, including IL-1, IL-6, and TNF-α, are primarily released from glial cells, especially microglia, and are implicated in depressive disorder development.^6^

Microglial cells, resident phagocytes in the central nervous system (CNS), play a crucial role as key responders to inflammatory processes. Upon activation, M1 microglia generate pro-inflammatory cytokines and mediators, whereas M2 microglia tend to produce anti-inflammatory cytokines, including transforming growth factor β (TGF-β) and IL-10. M2 microglia express various receptors associated with inhibiting inflammation and restoring homeostasis, including the mannose receptor, cluster of differentiation 206 (CD206).^7^^,^^8^ The imbalances between M1 and M2 microglia are thought to contribute to neuronal dysfunction and monoamine deficits, leading to MDD.^9^^,^^10^

Environmental stresses, including acute and chronic forms, are frequently employed to induce depression-like behaviors in animals. Common methods include the Forced Swim Test (FST)^11^ and the unpredictable chronic mild stress (UCMS)^12^ models. The FST gauges antidepressant-like effects by measuring rodent immobility in a water-filled cylinder, making it a widely used tool for rapid antidepressant screening. In contrast, UCMS exposes animals to various unpredictable mild stressors over an extended period, eliciting emotional irritabilities and abnormal phenotypes such as altered locomotion and reduced exploration behaviors in the open field test (OFT). Assessing depression-like behaviors in UCMS often involves the sucrose preference test (SPT), where decreased sucrose consumption reflects diminished “interest” in animals. This decline in sucrose preference, akin to anhedonia, a key symptom of human depression,^13^ is commonly observed in UCMS studies.

The UCMS model effectively replicates various depression-like symptoms, providing a valuable avenue for unraveling the molecular mechanisms underlying depression.^14^ Research indicates that UCMS induces elevated oxidative stress and pro-inflammatory signaling in the brain, leading to aberrant depression-like behaviors in animals.^15^^,^^16^ Consequently, this model exhibits strong validity and reliability for studying the effects of antidepressants.

Traditional Chinese medicine (TCM) is often employed as an adjunctive therapy for depression, with anti-inflammatory mechanisms being a key focus of research.^17^^,^^18^ *Cordyceps militaris* (Linn.) Link (CM) and *Armillaria mellea* (Vahl) P. Kumm. (AM) are both recognized for their anti-inflammatory properties, suggesting their potential in depression management.^19^, ^20^, ^21^ Previous studies have shown the antidepressant-like effects of CM and AM individually in animal models of depression.^22^^,^^23^

Although we have demonstrated the antidepressant-like effects of CM and AM individually in the UCMS model and confirmed their anti-inflammatory mechanisms, their specific impact on microglia activation regulation remains unclear. Despite numerous medicinal mushroom formulae are available today, little is understood about the formulation of CM and AM regarding their antidepressant-like and immunomodulatory effects. Additionally, the effective doses of CM and AM extracts typically range in several hundred milligrams. TCM is often administered as a decoction or formula for optimized effects. In a prior study, we evaluated the combined CM and AM formula in our initial FST investigation and noted its antidepressant-like effects in that acute screening model (data not shown). Hence, this study aims to investigate whether combining CM and AM in a formula exhibits antidepressant-like effects in the UCMS model in a lower dosage range and to clarify the mechanism by which the CM-AM formula modulates activated microglia.

## 2 Materials and methods

### 2.1 Preparation and formulation of CM and AM extracts

The CM and AM extracts used in this study were provided by the Taiwan Agricultural Research Institute (TARI), Council of Agriculture (Taichung, Taiwan). The authentication, cultivation, rhizomorph formation and preparation of the CM and AM extracts were as described previously.^22^^,^^23^ In brief, the AM strain was cultured on potato dextrose agar medium for 14 days, then transferred to a flask containing malt extract, glucose, and wheat extract. Following shake-flask fermentation for 7 days, and subsequent static incubation for 14 days, the culture was lyophilized. To obtain the AM extract, the dried powder underwent aqueous extraction (20 times at 100 °C for 1 h), followed by filtration and freeze-drying. As for the CM strain, it was cultured on potato dextrose agar medium for 14 days. Mycelial agar discs (5-mm diameter) were then transferred to potato dextrose broth in flasks and incubated in darkness for 5 days on a rotary shaker. Brown rice and water were sterilized in glass bottles, inoculated with liquid spawn until mycelial colonization. Primordia formation was induced by illuminating the room for 12 h daily. Mature fruiting bodies were harvested and lyophilized. To produce the CM extract, 100 g of dried powder underwent aqueous extraction (15 times at 100 °C for 1 h), followed by filtration and freeze-drying to yield a fine powder.

Based on our preliminary FST (data not shown), the formulation combining CM (15.6 mg/kg bw/day) and AM (31.3 mg/kg bw/day) showed the highest efficacy in rescuing depression-like behaviors in the acute rodent model of depression. The formulations used in the animal behavioral studies were categorized as follows: low dose (LD) formulation (7.8 mg/kg bw/day CM and 15.7 mg/kg bw/day AM), medium dose (MD) formulation (15.6 mg/kg bw/day CM and 31.3 mg/kg bw/day AM), and high dose (HD) formulation (31.2 mg/kg bw/day CM and 62.6 mg/kg bw/day AM). All formulations with varying extract concentrations were prepared in the aqueous solution specified for the experiment.

### 2.2 Measurements of the bioactive components of the CM-AM formula

The analysis of adenosine, cordycepin, and mannitol in the CM-AM formula was performed using a high-pressure liquid chromatography (HPLC) system. This system included a Waters Alliance HPLC 2695 equipped with either a 2996 UV/VIS or a 2414 RI detector (Milford, MA, USA). For armillarisin A analysis, a Liquid Chromatograph/Mass Spectrometer/Mass Spectrometer (LC/MS/MS) system was utilized. This system featured a Shimadzu LC-20AD quaternary pump, a Shimadzu SPD-M20 photodiode array detector, and a Shimadzu LCMS-804 mass detector. The analyses of bioactive components were carried out at Herbioteck CO., LTD., Taipei, Taiwan. Standards of adenosine, cordycepin, mannitol, and armillarisin A were procured from Sigma Chemical Corporation. Analytical methods followed the Herbioteck protocols WI-504-800-79 (adenosine and cordycepin), WI-504-800-80 (mannitol), and WI-504-800-81 (armillarisin A). The HPLC chromatograms of each standard and the CM-AM formula were shown in Supplementary Figs. 1 and 2, respectively. The LC/MS/MS profiles of armillarisin A standard and CM-AM formula was shown in Supplementary Fig. 3. The amounts of major components including adenosine, cordycepin, mannitol and armillarisin A in the CM-AM formula were shown in Table 1.

**Table 1:** The amount of active compounds of the CM-AM^a^ formula.

	Adenosine (μg/mL)	Cordycepin (μg/mL)	Mannitol (mg/mL)	Armillarisin A (ppb)
**The CM-AM formula**	8.69 ± 1.14	13.28 ± 0.53	62.17 ± 0.51	0.07 ± 0.00

a , *Cordyceps militaris* (Linn.) Link (CM) and *Armillaria mellea* (Vahl) P. Kumm. (AM).

### 2.3 Experimental animals

Seventy-two male Sprague-Dawley (SD) rats, aged 4 weeks, were procured from the BioLASCO Taiwan Co., Ltd (Ilan, Taiwan). These rats were individually raised in cages in the Animal Facility, Institute of Food Science and Technology, National Taiwan University. The rats were maintained under standard environmental conditions, including a temperature of 22 ± 2 °C, relative humidity ranging from 50 to 60 %, and a light-dark cycle of 12 h. Water and a rodent chow diet were made available *ad libitum*, allowing the rats unrestricted access. Food and water consumption, along with the body weight of the rats, were recorded on a weekly basis. All procedures related to animal handling and housing adhered to institutional guidelines and were approved from the local Institutional Animal Care and Use Committee (IACUC) of National Taiwan University, Taiwan (Approval Number: NTU107-EL-00143).

### 2.4 Groups subjected to different treatments

Following a 2-week acclimatization period, rats were then randomly assigned to six groups (n = 12 per group) as follows: control group (CTL), negative control group (NCTL), positive control group (PCTL), low dose CM-AM formulation group (LD), medium dose CM-AM formulation group (MD), and high dose CM-AM formulation group (HD). The interventions were orally delivered (*via* gavage) daily for a duration of 4 weeks in the UCMS model. In the PCTL group, a positive antidepressant, fluoxetine hydrochloride (10 mg/kg bw/day, dissolved in a 0.5 % carboxymethylcellulose (CMC) aqueous solution), was utilized and sourced from China Medical University Hospital (Taichung, Taiwan). The CTL and NCTL groups were administered solely with the 0.5 % CMC solution. The formulation compositions and extract concentrations of LD, MD and HD groups were described in Materials and Methods 2.1.

### 2.5 Unpredictable chronic mild stress (UCMS)

The UCMS procedure was conducted in accordance with the methods outlined in our prior study.^17^ Rats undergoing the UCMS paradigm were exposed to multiple stressors, including: food deprivation (24 h), water deprivation (24 h), empty drinking bottles (1 h), cage tilting at 45° (18 h), moist bedding (18 h), disruption of the light-dark cycle (2 h each time, three times daily), continuous illumination or dark (24 h), restricted access to food (a small amount of food was placed, 2 h), cold water swimming (4 °C, 5 min), physical restraint (2 h) and white noise (100 dB, 3 h). Stressors were randomly applied 1–2 times per week to ensure the unpredictability and prevent acclimatization during the 4-week-long stress model. The rats within the CTL group were not disturbed and were kept in a dedicated, stress-free room throughout the entire experiment. A summarization of each group subjected to different treatments and with or without the UCMS procedure was shown in Table 2.

**Table 2:** A summarization of the treatments, procedures, and behavioral tests of each group in this study.

	CTL^a^	NCTL^b^	PCTL^c^	LD^d^	MD^e^	HD^f^
**Treatment**	0.5 % CMC^g^	0.5 % CMC	10 mg/kg bw/day fluoxetine hydrochloride	7.8 mg/kg bw/day CM^h^ and 15.7 mg/kg bw/day AM^i^	15.6 mg/kg bw/day CM and 31.3 mg/kg bw/day AM	31.2 mg/kg bw/day CM and 62.6 mg/kg bw/day AM
**Procedure**	Undisturbed	UCMS^j^	UCMS	UCMS	UCMS	UCMS
**Behavioral tests**	SPT^k^ and OFT^l^	SPT and OFT	SPT and OFT	SPT and OFT	SPT and OFT	SPT and OFT

a Control group (CTL).

b Negative control group (NCTL).

c Positive control group (PCTL).

d Low-dose formulation group (LD).

e Medium-dose formulation group (MD).

f High-dose formulation group (HD).

g Carboxymethylcellulose (CMC).

h *Cordyceps militaris* (Linn.) Link (CM).

i *Armillaria mellea* (Vahl) P. Kumm. (AM).

j Unpredictable chronic mild stress (UCMS).

k Sucrose preference test (SPT).

l Open field test (OFT).

### 2.6 Sucrose preference test (SPT)

The SPT was carried out at the end of every week during the UCMS manipulation as described in our previous works.^17^^,^^22^^,^^23^ Prior to the commencement of UCMS, each rat was individually placed in a cage with a single bottle containing 1 % sucrose solution for a 24-h adaptation period. Subsequently, another bottle containing water was introduced for an additional 24 h. After the adaptation phase, the rats underwent a 24-h period of water and food deprivation before the SPT was conducted. During the SPT, rats were given access to two bottles containing a 1 % sucrose solution and water. After 1 h, the amounts of consumed sucrose solution and water were recorded. The sucrose preference was determined using the formula: sucrose preference (%) = sucrose intake in grams/[sucrose intake in grams + water intake in grams] × 100.

### 2.7 Open field test (OFT)

The OFT was performed as described in our previous study.^17^ In short, every rat was placed alone in the central area of an open field apparatus with a black floor and permitted to explore the various sections of the apparatus. The behavior of each rat was captured on video for a duration of 5 min. The total traveled distance, number of crossings, and time in the center area were analyzed using TopScanLite™ 2.0 software (CleverSys Inc, Reston, VA, U.S.A.). The OFT was carried out on the 28th day, the last day of the entire experiment.

### 2.8 Tissue preparation

Upon the end of the experiment, rats were euthanized by CO_2_ and decapitated immediately. The prefrontal cortex and hippocampus were promptly dissected using the method described by Glowinski and colleagues,^24^ with minor adjustments and frozen at −80 °C for subsequent analysis. The blood was collected at the time of decapitation. The serum was isolated by subjecting it to a centrifuge at 3000×*g* and 4 °C for 20 min and preserved at −80 °C for subsequent analysis. For immunohistochemistry (IHC) studies, rats were euthanized by CO_2_ and transcardially perfused. Tissues were cleared with 200 mL of a phosphate buffered saline (PBS) solution (pH 7.4) and subsequently fixed using 200 mL of 4 % paraformaldehyde (pH 9.6) in PBS. Brains were dissected out and placed into the same fixative solution overnight for post-fixing. Fixed brains were sent to CIS Biotechnology (Taichung, Taiwan) for histological processing, embedded in paraffin, and underwent serial coronal sections sliced at 5 μm. The hippocampal areas (Bregma −2.38 to −3.01 mm) were selected for IHC studies.

### 2.9 Enzyme-linked immunosorbent assay (ELISA) analysis for serum corticosterone

The serum corticosterone levels were assessed utilizing a corticosterone ELISA kit (Enzo Life Science Inc., NY, USA) as per the manufacturer's instructions.

### 2.10 Quantification of monoamine neurotransmitters in the prefrontal cortex and hippocampus

The quantification of monoamines was performed as described in our previous works.^17^^,^^25^ In brief, the prefrontal cortex and hippocampus were homogenized with chilled homogenizing solution. The samples were then centrifuged, filtered, and subjected to high performance liquid chromatography (HPLC, L-7100 pump, Hitachi Co. Ltd., Tokyo, Japan) coupled with an electrochemical detector (ECD, DECADE Elite, Antec Scientific, NV Zoeterwoude, Netherlands) and a C18 column (SPOLAR C18 S5 column, 4.6 mm I.D. × 250 mm, 5 μm, OSAKA SODA CO., LTD., Osaka, Japan). The concentration of serotonin (5-HT), 5-hydroxyindoleacetic acid (5-HIAA), dopamine (DA) and 3,4-dihydroxyphenylacetic acid (DOPAC) were analyzed. The turnover rates of 5-HT and DA were determined as percentages, calculated as 5-HIAA/5-HT (%) and DOPAC/DA (%), respectively.

### 2.11 Immunohistochemical staining for Iba-1-positive microglia in the hippocampus

Paraffin-embedded tissues were sliced into sections of 5 μm thickness and placed on glass slides. All section were subjected to a 60 °C heating process for a duration of 20 min and placed in a xylene solution. Sections were then rehydrated in a series of graded ethanol followed by 100 %, 95 %, 85 %, 75 % and 50 %. Sections were placed in the Trilogy™ buffer (Sigma, St. Louis, MO, U.S.A) in a pressure cooker at high pressure and temperature for 15 min for antigen retrieval. Next, sections were incubated with Hydrogen Peroxide Block (BioTnA, Kaohsiung, Taiwan) for 20 min and with Immunoblock (BioTnA, Kaohsiung, Taiwan) for 1 h at 25 °C to prevent nonspecific binding. The primary antibody, rabbit anti-Iba-1 (#019–19741, FUJIFILM Wako Chemicals, 1:600), was reconstituted in PBS with Tween (PBST) and incubated with sections for 2 h at 25 °C. Sections were washed with PBST and incubated with mouse/rabbit probe HRP labeling kit (#TAHC03D-15, BioTnA, Kaohsiung, Taiwan) for 30 min at 25 °C, and washed with PBST. Sections were incubated with 3,3′-diaminobenzidine (#TAHC03D-15, BioTnA, Kaohsiung, Taiwan) for 5 min at 25 °C. The reaction was terminated by adding PBS. Finally, sections were mounted with coverslips using the mounting medium, Micromount (Leica Biosystems, Germany).

### 2.12 Image analysis of Iba-1-positive microglia in the hippocampus

Image analysis of the Iba-1-positive microglia morphology was performed based on the work done by Morrison and colleagues^26^ with modifications. Images were obtained from Stereo Investigator® (MBF Bioscience, Williston, VT, U.S.A.) using a 60× objective. Three coronal hippocampus sections per animal were imaged, and ten cells per brain were selected from each animal. The morphology of hippocampal microglia was subjected to skeleton analysis and fractal analysis. Series of steps were conducted by using ImageJ (https://imagej.nih.gov/ij/) to convert photomicrographs to binary images. The images were filtered to soften the background, enhance the contrast, and transformed into the 8-bit grayscale, binarized, black and white images. The processed images were then cropped and saved separately for the skeleton and fractal analyses. The microglia selection and image analysis were performed independently by at least two researchers under de-identification.

### 2.13 Skeleton analysis

The Analyze Skeleton Plugin (http://imagej.net/AnalyzeSkeleton) was employed to skeletonize and analyze microglia. This plugin tagged skeletal features of the microglia that were relevant to microglia ramification. The output of the skeleton analysis was the number of endpoints in each cell (microglia).

### 2.14 Fractal analysis

The fractal analysis (FracLac for ImageJ) was performed to quantify the microglia complexity (fractal dimension, D_B_). FracLac used a box plot protocol to assess the D_B_ for individual cells (microglia), determining the level of pixel detail as the scale increases. The equation used in the analysis was the regression slope ln(N)/ln(*ε*), where N = the number of pixels or “detail” at a particular scale (ɛ). For more information related to these calculations and relationships, refer to the provided reference guide for FracLac in ImageJ.

(http://rsb.info.nih.gov/ij/plugins/fraclac/FLHelp/Introduction.htm) and consult additional references^27^,^28^. The fractal analysis output was fractal dimension (D_B_, ranging from 1.0 to 2.0) in each cell (microglia), which was interpreted as the cell complexity and the cell shape.

### 2.15 Extraction of proteins and Western blot analysis

The methods of protein extraction and Western blot analysis were followed the procedures outlined in our prior investigations.^22^^,^^25^ The primary antibodies employed were as follows: rabbit anti-IL-1β (#R30418, NSJ Bioreagents, 1:1000), mouse anti-TNFα (#52B83, Santa Cruz Biotech, 1:1000), rabbit anti-IL-10 (#R30118, NSJ Bioreagents, 1:1000), rabbit anti-TGF-β (#3711, Cell signaling, 1:1000) rabbit anti-mannose receptor (CD206) (#ab64693, Abcam, 1:1000) rabbit anti-GAPDH (#5174, Cell signaling, 1:3000). The secondary antibodies utilized were goat anti-rabbit IgG HRP-linked antibody (#7074, Cell signaling, 1:5000) and horse anti-mouse IgG HRP-linked antibody (#7076, Cell signaling, 1:5000). The detection of immune complexes was performed using the UVP Biospectrum AC system (UVP, U.S.A).

### 2.16 Statistical analysis

Results are presented as mean ± SD. The data were analyzed using one-way analysis of variance (ANOVA) followed by post-hoc Tukey's multiple comparisons test. A value of *p* < 0.05 was considered significant. All statistical analyses and graphs were carried out by GraphPad Prism 10 (La Jolla, CA).

## 3 Results

### 3.1 Effects of the CM-AM formula on body weight, food and water intakes

**Fig. 1:**
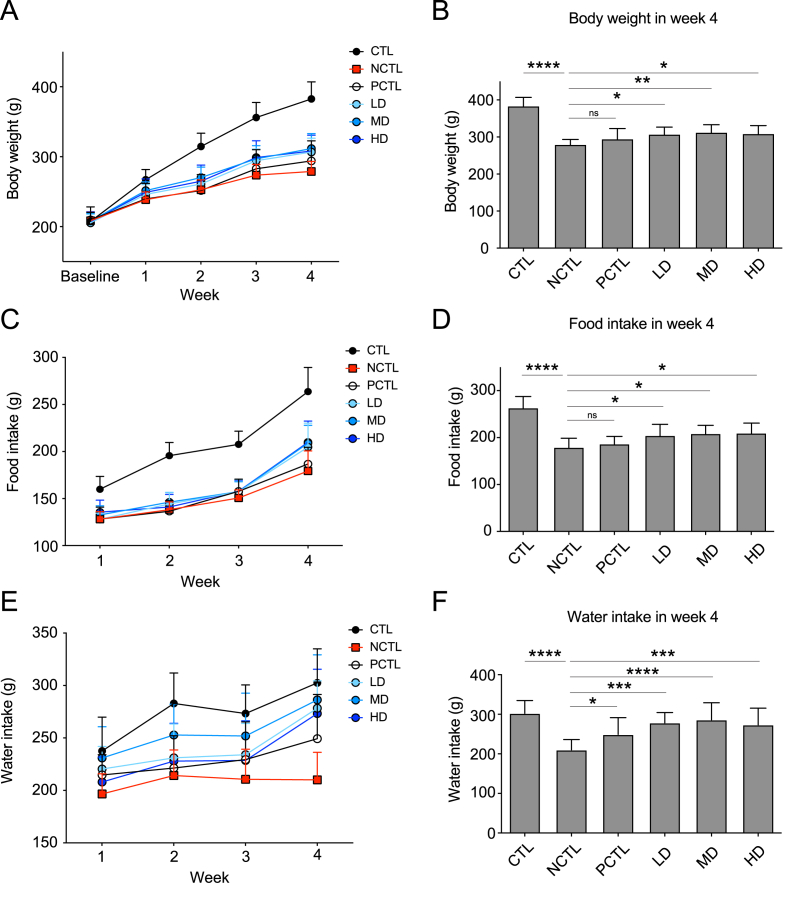
Impacts of the CM-AM formula treatments on body weight and daily intakes throughout the UCMS paradigm The weekly body weight (A), food intake (C), water intake (E) during the UCMS paradigm. The body weight (B), food intake (D) water intake (F) in week 4. The data are presented as mean ± SD with a sample size (n) of 12. Subsequently, ANOVA and Tukey's test were applied. *p < 0.05; **p < 0.01; ***p < 0.001; ****p < 0.0001; ns, no significance.

To evaluate the antidepressant-like effects of the CM-AM formula, we subjected 6-week-old rats to a 4-week UCMS paradigm. Baseline body weights were recorded before the paradigm initiation. Throughout the UCMS experiment, we measured body weights (Fig. 1A), food intake (Fig. 1C), and water intake (Fig. 1E) weekly. Following the 4-week UCMS procedure, rats in the NCTL group exhibited significantly lower body weight compared to the CTL group (Fig. 1B). However, all doses of the CM-AM formula (LD, MD, HD) significantly mitigated this decrease in body weight gain, except for the PCTL group (Fig. 1B). Additionally, UCMS significantly reduced food and water intake throughout the experiment (Fig. 1C and E) and specifically in week 4 (Fig. 1D and F). Similarly, rats receiving any dose of the CM-AM formula significantly alleviated the decrease in food and water intake compared to the NTCL group (Fig. 1D and F). Food intake in the PCTL group remained lower compared to the NCTL group, consistent with the observed decrease in body weight. Notably, water intake in the PCTL group was higher than that of the NCTL group (Fig. 1F). These findings suggest that the CM-AM formula could serve as an alternative approach for managing depression with minimal side effects.

### 3.2 Administration of the CM-AM formula restored the depressive behaviors in the sucrose preference test

**Fig. 2:**
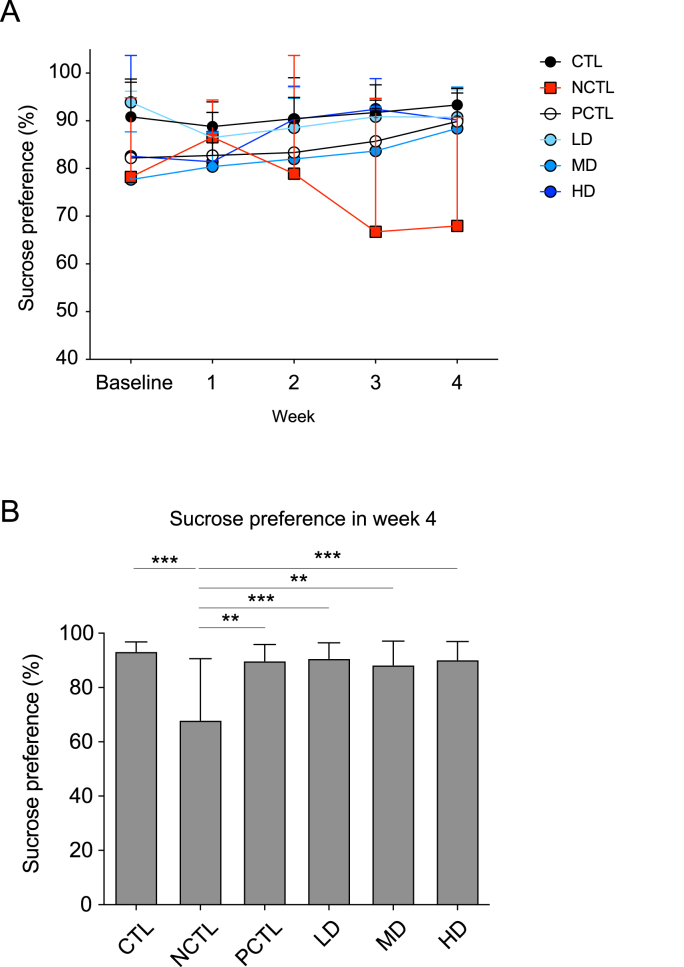
Impacts of the CM-AM formula treatments on the sucrose preference in the UCMS model The weekly sucrose preference throughout the UCMS experiment (A). The sucrose preference in week 4 (B). The data are presented as mean ± SD with a sample size (n) of 12. Subsequently, ANOVA and Tukey's test were applied. **p < 0.01; ***p < 0.001.

To gauge the initiation of depressive behaviors resembling those seen in depression, such as anhedonia, (a reduction in interest to consume sucrose solution), the SPT was employed. As depicted in Fig. 2A, the sucrose preference of each group was recorded weekly throughout the experiment. Rats in the NCTL group exhibited a decreasing trend in sucrose preference starting from week 1, reaching a significant difference compared to the CTL group in week 4 (Fig. 2B). This decline in sucrose preference indicated the successful induction of anhedonia, a critical depression-like behavior in rodents, in our UCMS paradigm. In contrast, the sucrose preferences of rats in the PCTL and all doses of the CM-AM formula (LD, MD, HD) groups were restored compared to those in the NCTL group (Fig. 2B). This restoration suggests that the administration of the CM-AM formula mitigated the depression-like behaviors observed in the SPT.

### 3.3 Administration of the CM-AM formula ameliorated the depression-like behaviors in the open field test

**Fig. 3:**
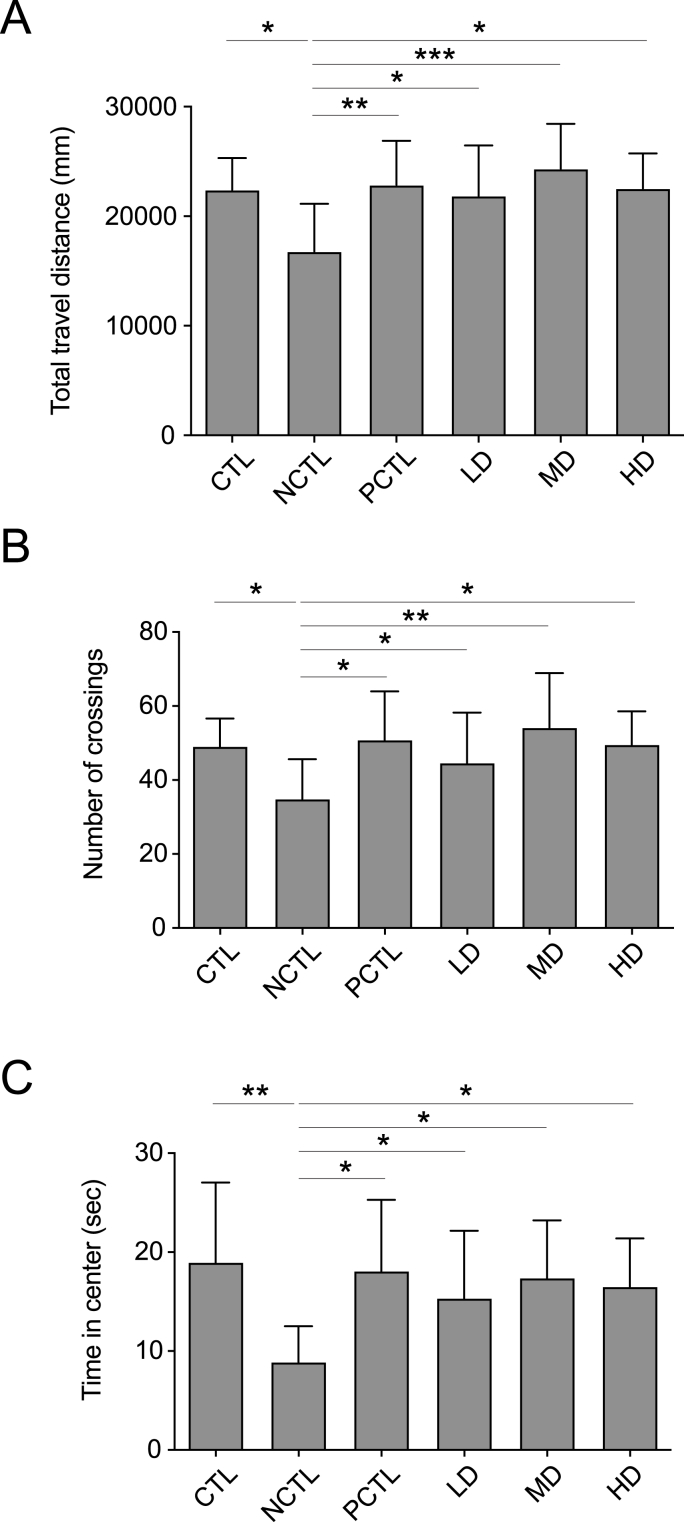
Impacts of the CM-AM formula treatments on the open field test in the UCMS model The total traveled distance (A), the number of crossing (B) and the time spent in the center (C) in the open field test at week 4 of the model. The data are presented as mean ± SD with a sample size (n) of 12. Subsequently, ANOVA and Tukey's test were applied. *p < 0.05; **p < 0.01; ***p < 0.001.

To corroborate the findings from the SPT, we subsequently utilized the OFT to assess depression-like behaviors, such as locomotor abnormalities, in UCMS-exposed rats. Parameters such as total traveled distance, number of crossings, and time spent in the center (Fig. 3) were measured to assess depressive tendencies. As depicted in Fig. 3A–C, reductions in these parameters were observed in rats from the NCTL group, indicative of depression-like behavior. Similarly, rats treated with fluoxetine (PCTL group) and all doses of the CM-AM formula (LD, MD, HD) exhibited significant reversals in the decreased total traveled distance, number of crossings, and time spent in the center (Fig. 3A–C). These findings collectively support the hypothesis that the CM-AM formula possesses antidepressant-like effects in UCMS-exposed rodents.

### 3.4 Administration of the CM-AM formula attenuated the stress-induced corticosterone elevation in serum

**Fig. 4:**
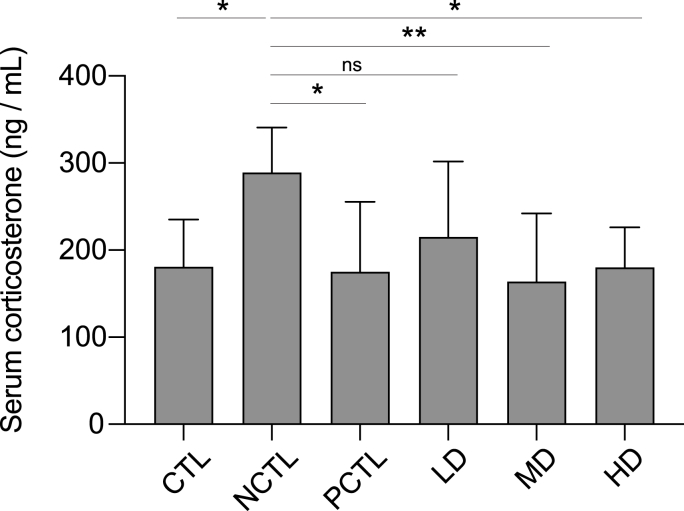
Impacts of the CM-AM formula treatments on corticosterone levels in the serum within the UCMS model The data are presented as mean ± SD with a sample size (n) of 8. Subsequently, ANOVA and Tukey's test were applied. *p < 0.05; **p < 0.01; ns, no significance.

Chronic stress disrupts the hypothalamic-pituitary-adrenal (HPA) axis, resulting in increased circulating corticosterone levels. We observed a significant elevation in serum corticosterone levels in the NCTL group compared to the CTL group (Fig. 4), indicating successful manipulation of stress hormone dysregulation through UCMS. Conversely, except for the LD of the CM-AM formula, fluoxetine treatment (PCTL group), MD and HD doses of the CM-AM formula effectively mitigated the elevated serum corticosterone levels in UCMS-exposed rats (Fig. 4). This suggests a potential regulatory effect of the CM-AM formula on the dysregulated HPA axis associated with chronic stress and depression.

### 3.5 Administration of the CM-AM formula normalized the turnover rates of monoamines neurotransmitters in the hippocampus and the prefrontal cortex

**Fig. 5:**
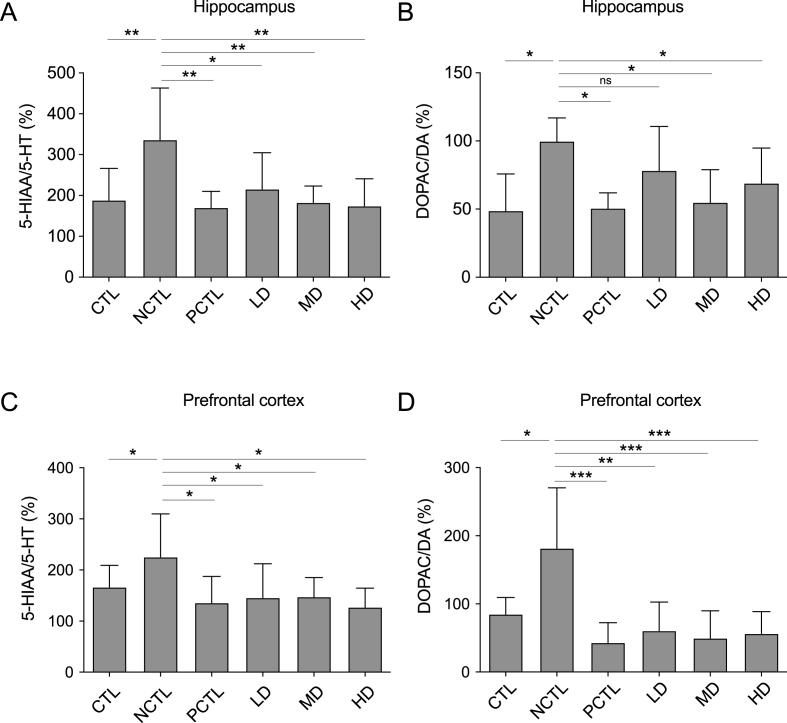
Impacts of the CM-AM formula treatments on the turnover rates of serotonin and dopamine in the rat brains under the UCMS paradigm The 5-HT turnover rates in the hippocampus (A) and the prefrontal cortex (C); the DA turnover rates in the hippocampus (B) and the prefrontal cortex (D) of the rats within the UCMS model. The data are presented as mean ± SD with a sample size (n) of 8. Subsequently, ANOVA and Tukey's test were applied. *p < 0.05; **p < 0.01; ***p < 0.001; ns, no significance.

The deficiency of cerebral monoamines is strongly associated with depressive disorders,^4^ exacerbated by elevated circulating corticosterone.^29^ We assessed levels of cerebral serotonin (5-HT), dopamine (DA), and their metabolites, 5-HIAA and DOPAC, respectively. Data on the turnover rates of both monoamines in the hippocampus and prefrontal cortex are presented as percentages (Fig. 5). In the hippocampus, turnover rates of 5-HT and DA were up-regulated in the NCTL group compared to the CTL group (Fig. 5A and B). Fluoxetine treatment (PCTL group) reversed these rates, as did MD and HD doses of the CM-AM formula (Fig. 5A and B). In the prefrontal cortex, similar patterns were observed, with higher turnover rates in the NCTL group, normalized by fluoxetine and all CM-AM formula doses (Fig. 5C and D). Once again, these results suggest that the CM-AM formula eliminates monoamine dysregulation induced by UCMS, indicating its antidepressant-like effect.

### 3.6 Administration of the CM-AM formula alleviated the stress-induced microglia activation in the hippocampus

**Fig. 6:**
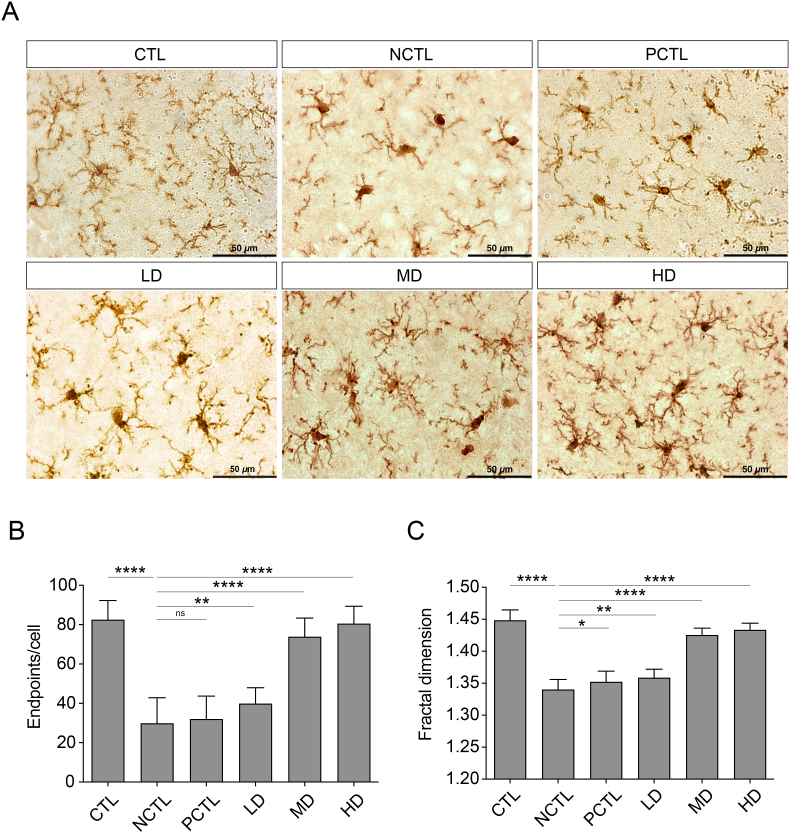
Impacts of the CM-AM formula treatments on morphologies of the hippocampal microglia in the UCMS model The representative images showing Iba-1-positive microglia in the hippocampus of the rats in the UCMS model (A). Scale bar: 50 μm. The morphological analysis of the hippocampal microglia ramification (B) and complexity (C) of the UCMS-induced rats. The data are presented as mean ± SD with a sample size (n) of 3 (n = 3, 30 microglial per group were analyzed). Subsequently, ANOVA and Tukey's test were applied. *p < 0.05; **p < 0.01; ****p < 0.0001; ns, no significance.

We next employed immunohistochemistry (IHC) to stain the Iba-1-positive microglia in the hippocampus to investigate inflammatory events in the CNS. The morphology of the hippocampal microglia in each group is shown in Fig. 6A. We quantified the microglia process endpoints and fractal dimension to assess cell ramification and cell complexity, respectively. In the CTL group, most microglia remained in a resting state with ramified cell bodies and multiple endpoints (Fig. 6A and B). Fractal dimension analysis revealed a complexity value (D_B_) of approximately 1.45 for resting microglia in the CTL group (Fig. 6A and C). However, in the NCTL group, both endpoints and the D_B_ of microglia significantly decreased, indicating changes in microglia morphology (Fig. 6A–C). These activated, proinflammatory microglia in the NCTL group exhibited an enlarged cell body, fewer processes, and a spherical shape (Fig. 6A). Conversely, rats in the PCTL and all doses of the CM-AM formula (LD, MD, HD) alleviated these morphological changes associated with pro-inflammation, except for rats in the PCTL group in terms of restoring the microglial endpoints (Fig. 6A–C). These results highlight that the CM-AM formula modulates the inflammatory response in the CNS induced by UCMS.

### 3.7 Administration of the CM-AM formula reversed the proinflammatory signaling pathways in the hippocampus

**Fig. 7:**
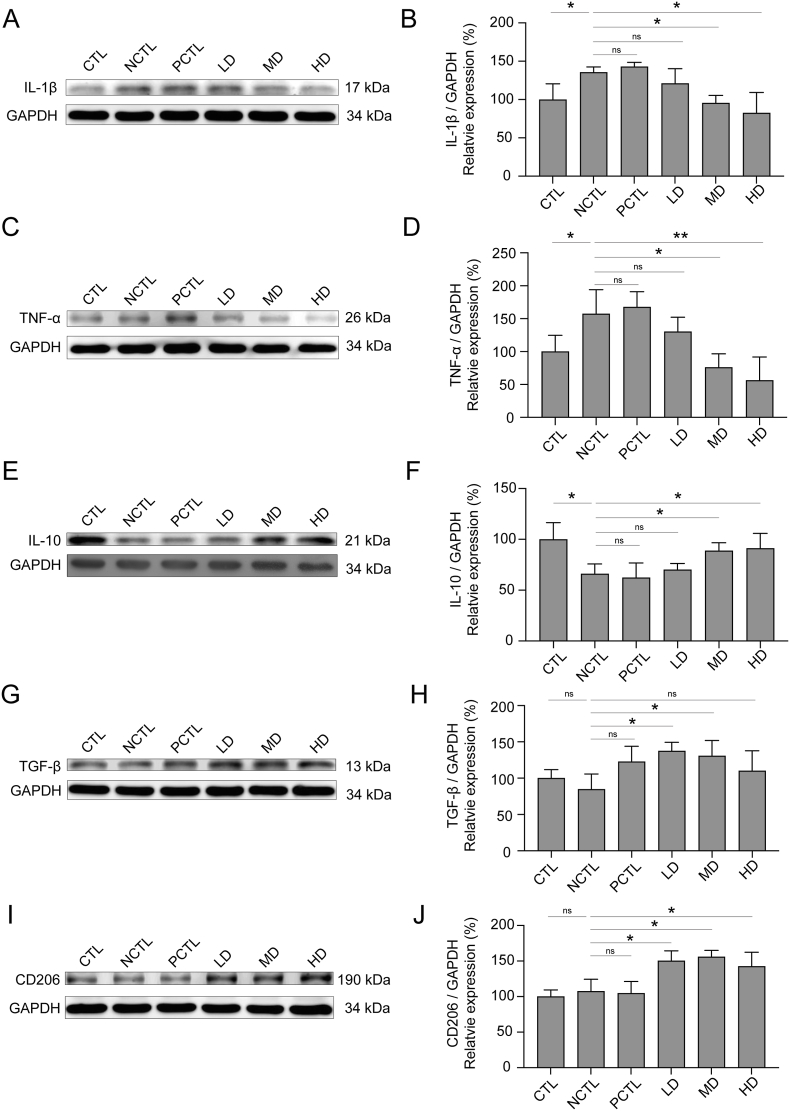
Impacts of the CM-AM formula treatments on pro- and anti-inflammatory proteins expressions in the rats hippocampus in the UCMS model Representative immunoblots of IL-1β (A), TNF-α (C), IL-10 (E), TGF-β (G) and CD206 (I) in hippocampal lysates prepared from rats in the UCMS model. Relative expression levels of IL-1β (B), TNF-α (D), IL-10 (F), TGF-β (H) and CD206 (J) in hippocampal lysates among groups under the UCMS manipulation. The data are presented as mean ± SD with a sample size (n) of 3. Subsequently, ANOVA and Tukey's test were applied. *p < 0.05; **p < 0.01; ns, no significance.

We further analyzed hippocampal proteins to investigate proinflammatory signaling. Consistent with our IHC findings, the NCTL group showed significantly elevated IL-1β and TNF-α protein levels compared to the CTL group, indicating UCMS-induced inflammation (Fig. 7A–D). Administration of MD and HD doses of the CM-AM formula significantly reduced IL-1β and TNF-α levels compared to the NCTL group (Fig. 7A–D), while neither the PCTL group nor LD of the CM-AM formula showed significant differences. We then examined anti-inflammatory cytokines in the hippocampus. The NCTL group exhibited lower IL-10 and TGF-β protein levels compared to the CTL group, indicating an imbalance in pro- and anti-inflammatory responses (Fig. 7E–H). MD and HD doses of the CM-AM formula reversed the lower IL-10 levels (Fig. 7E and F), and LD and MD doses improved the lower TGF-β levels compared to the NCTL group (Fig. 7G and H). Despite higher proinflammatory and lower anti-inflammatory cytokine expressions, there was no significant difference in CD206 (one of the markers of M2 microglia) protein expression between the NCTL, CTL, and PCTL groups (Fig. 7I and J). Notably, all CM-AM formula doses significantly increased CD206 protein levels compared to the NCTL group (Fig. 7I and J). These findings suggest that the CM-AM formula downregulates proinflammatory cytokine expressions, upregulates anti-inflammatory signaling pathways, and balances M1/M2 microglia ratios in the hippocampus, potentially contributing to its antidepressant-like effects.

## 4 Discussion

In previous studies, we showed that water extracts of CM and AM effectively prevented depression-like behaviors in rodent models exposed to chronic stress.^22^^,^^23^ The doses administered ranged in the hundreds of milligrams. In this study, we investigated the antidepressant-like effects and potential mechanisms of combining these mushroom extracts in a CM-AM formula using a UCMS animal model of depression. We found that the effective doses of the CM-AM formula were in the range of dozens of milligrams, indicating a potential enhancement when these ingredients are combined.

The 4-week UCMS paradigm effectively induced depression-like behaviors, such as growth retardation, reduced food and water intake, anhedonia, and decreased locomotor activities. Consistent with our previous studies,^22^^,^^23^ the CM-AM formula reversed these declines in daily intake and impaired weight gain. Studies have also shown that administering CM or AM extracts to animals did not affect weight gain or food intake, ruling out hyperphagia as a factor.^30^^,^^31^ Importantly, the CM-AM formula exhibited fewer side effects compared to the conventional antidepressant fluoxetine in restoring body weight and food intake, suggesting its potential as a novel therapy. Furthermore, no signs of liver dysfunction side effects, such as elevated serum levels of alanine aminotransferase (ALT) and aspartate aminotransferase (AST), were observed in rats treated with the CM-AM formula (data not shown). Assessing the safety profile of the CM-AM formula and establishing its no-observed-adverse-effect level (NOAEL) is crucial for preempting potential side effects in future applications.

Anhedonia, characterized by reduced responsiveness to natural rewards, often leads to a decline in the consumption of enjoyable foods and sweet beverages.^13^ Our findings indicate that both fluoxetine and the CM-AM formula reversed the decreased sucrose preference induced by UCMS. Additionally, we utilized the OFT to confirm depression-like behaviors in rodents,^32^ assessing autonomous behaviors as depression-like phenotypes.^12^ Rats subjected to 4 weeks of UCMS manipulation displayed hypoactivity and reduced interest in exploration. Consistent with the SPT results, administration of fluoxetine and the CM-AM formula reversed the decreased total traveled distance, number of crossings, and time spent in the center. These behavioral outcomes align with our previous studies^22^^,^^23^ and others,^33^, ^34^, ^35^ suggesting that the CM-AM formula could serve as a reliable and effective complementary treatment for depression.

In animal experiments, sustained corticosterone injections induce depression-like behaviors in rodents,^36^ while antidepressant treatment reduces circulating corticosterone levels in chronic stress models.^37^ Our findings indicate that the CM-AM formula decreased elevated serum corticosterone levels in the UCMS model, suggesting its ability to regulate stress-response systems in animals. Elevated corticosterone levels can disrupt the availability of monoamine neurotransmitters by affecting metabolic enzyme synthesis.^29^ Studies have shown that rats subjected to UCMS manipulation exhibit higher turnover rates or lower levels of cerebral 5-HT and DA.^17^^,^^22^^,^^25^^,^^38^ Similarly, 5-HT and DA turnover rates were significantly increased in both the prefrontal cortex and hippocampus, indicating insufficient cerebral monoamines due to chronic stress. However, treatment with both fluoxetine and the CM-AM formula normalized the upregulated turnover rates of cerebral 5-HT and DA. Thus, our results suggest that the regulation of cerebral neurotransmitters is one of the mechanisms underlying the antidepressant-like effects of the CM-AM formula.

Microglia are primary responders to infections and injuries in the CNS.^39^ M1 microglia produce pro-inflammatory cytokines (IL-1β, IL-6, and TNF-α), leading to blood-brain barrier (BBB) destruction, inflammatory cell influx, and neuronal damage. Conversely, M2 microglia secrete anti-inflammatory cytokines and mediators like brain-derived neurotrophic factor (BDNF), insulin-like growth factor-1 (IGF-1), TGF-β, and IL-10 to counter neuroinflammation and repair CNS damage.^40^ The imbalance between M1 and M2 microglia is critical in neuroinflammation and is considered a pathological contributor to depression.^9^ The morphological changes of microglia are closely linked to their role in inflammation regulation.^41^^,^^42^ Microglia with a ramified morphology are typically considered inactive and may engage in phagocytosis and neurotransmitter modulation.^43^ Upon activation, microglial cell bodies adopt a more spherical shape with a reduced number of processes.^44^ Additionally, activated microglia express higher levels of ionized calcium-binding adapter molecule (Iba-1) protein, which serves as an accurate indicator of microglia activation.^45^

Our results show that microglia in stressed rats exhibited activated M1 phenotypes, characterized by condensed cell bodies with fewer processes and lower fractal dimension features. Administration of the CM-AM formula, but not fluoxetine, restored these activated microglia morphologies. Additionally, Western blot results revealed disrupted and unbalanced levels of both pro-inflammatory and anti-inflammatory cytokines in the brains of rats exposed to chronic stress. Treatment with the CM-AM formula, but not fluoxetine, reduced pro-inflammatory cytokines and increased anti-inflammatory cytokines in the hippocampus. Interestingly, UCMS did not reduce the protein expression of CD206, the M2 microglial marker, indicating that chronic stress may disrupt the M1/M2 balance by increasing the dominance of M1 microglia. However, administration of the CM-AM formula increased CD206 protein levels.

Numerous active compounds have been identified in literature to support the anti-inflammatory action of edible mushroom extracts. Examples include polysaccharides, adenosine, ergothioneine, armillarisin A, cordycepin, and mannitol.^46^, ^47^, ^48^ Notably, armillarisin A has been shown to reduce IL-1β and increase IL-4 (an anti-inflammatory cytokine) levels in ulcerative colitis.^49^ Cordycepin is reported to attenuate lipopolysaccharide (LPS)-induced microglial activation, as evidenced by blocking nuclear factor kappa B (NF-κB) and down-regulating TNF-α and IL-1β expressions.^46^^,^^49^ Similarly, in a rat model of intracerebral hemorrhage, mannitol treatment is capable of regulating the balance of M1/M2 microglial activation, suggesting its immunomodulatory effect in cerebral injury.^50^ In the CNS, adenosine serves not only as a neuromodulator but also as a regulator for inflammation, implicated in chronic inflammatory pathologies, neurodegenerative diseases, and cancer.^51^ The adenosinergic system is thought to play a role in mood disorders, such as anxiety. Intriguingly, administration of adenosine has been shown to exhibit antidepressant effects by activating the adenosine A1 receptor. Together, this evidence is consistent with our present findings and previous works,^22^^,^^23^ supporting the anti-inflammatory action and neurotransmitters regulations of the CM-AM formula in managing depressive disorders.

Regarding the dose-response relationship of the CM-AM formula, similar to our prior discovery,^22^ we observed that the HD group does not demonstrate significantly greater effects compared to the MD group. This implies that the CM-AM formula may exhibit a U-shaped activity for its antidepressant-like effect. Overall, our findings suggest that the CM-AM formula modulate neurotransmissions, regulate inflammatory imbalance by down-regulating pro-inflammatory cytokines, up-regulating anti-inflammatory cytokines, and normalizing M1/M2 microglia dynamics, thereby exerting antidepressant-like effects.

## 5 Conclusion

Our study demonstrates that the CM-AM formula alleviated depression-like behaviors in the UCMS model, with effective doses in the range of dozens of milligrams. The antidepressant-like effect appears to involve neurotransmitter regulation and immunomodulation, as evidenced by the inhibition of pro-inflammatory cytokines, enhancement of anti-inflammatory mediators, and the balancing of M1 and M2 microglia fractions in the hippocampus. These findings support the potential of combining CM and AM in the formula as a complementary and alternative therapy for managing depressive disorders.

## Funding

The research was financially supported by the Taiwan Agricultural Research Institute (TARI) under grants 107A3350 and 108A3302-1.

## Author contributions

YEL, conceptualization, investigation, data curation; formal analysis, visualization, writing-original draft, writing-review and editing.

HPL, investigation, data curation, formal analysis, visualization, manuscript editing.

KHL, project administration, validation, funding acquisition, manuscript editing.

YJH & SP, formal analysis, manuscript editing.

WTL, YSL & MHC, resource, manuscript editing.

LYS, supervision, funding acquisition, writing original draft, writing-review and editing.

## Declaration of competing interest

The authors declare that they have no conflicts of interest to disclose.
