# Supplementary material for: Cordyceps militaris and Armillaria mellea formula alleviates depressive behaviors via microglia regulation in an unpredictable chronic mild stress animal model
Source: J Tradit Complement Med. 2024 May 16;15(1):24–35. doi: 10.1016/j.jtcme.2024.05.003 (PMC11725130; doi:10.1016/j.jtcme.2024.05.003)
Supplement: Multimedia component 1 [file mmc1.docx]

**Supplementary data**

A


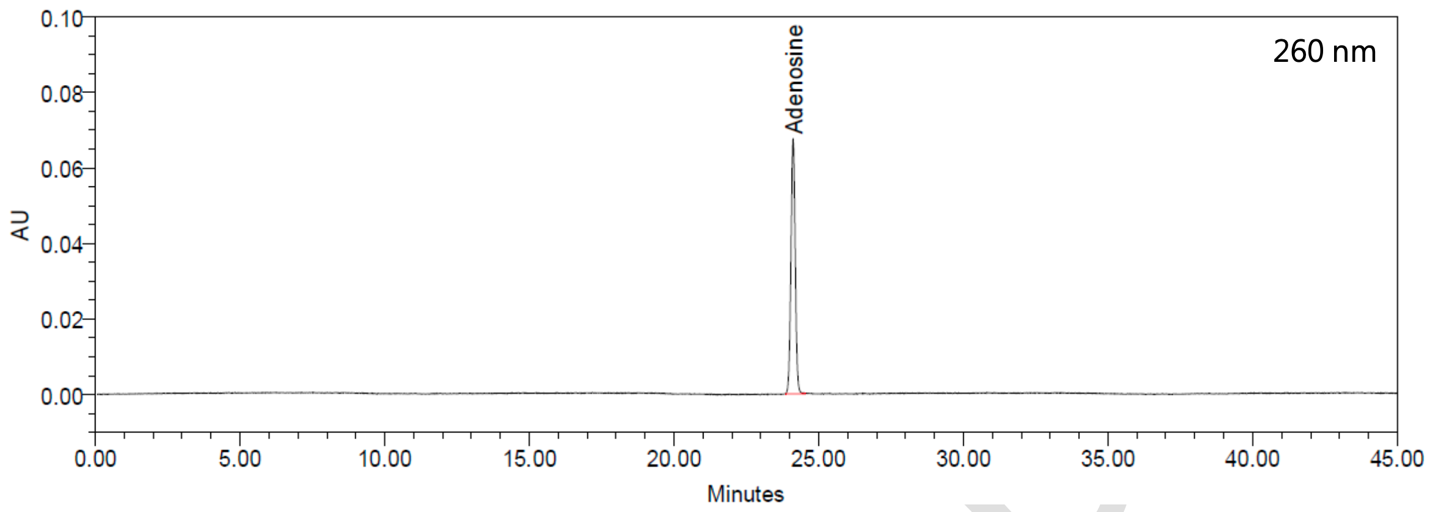


B


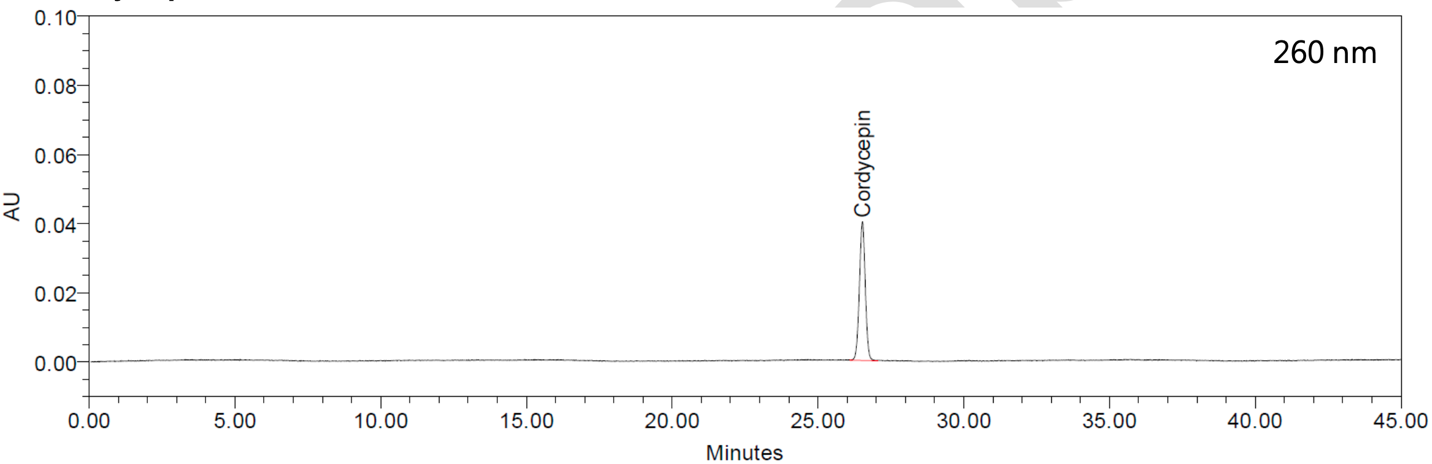


C


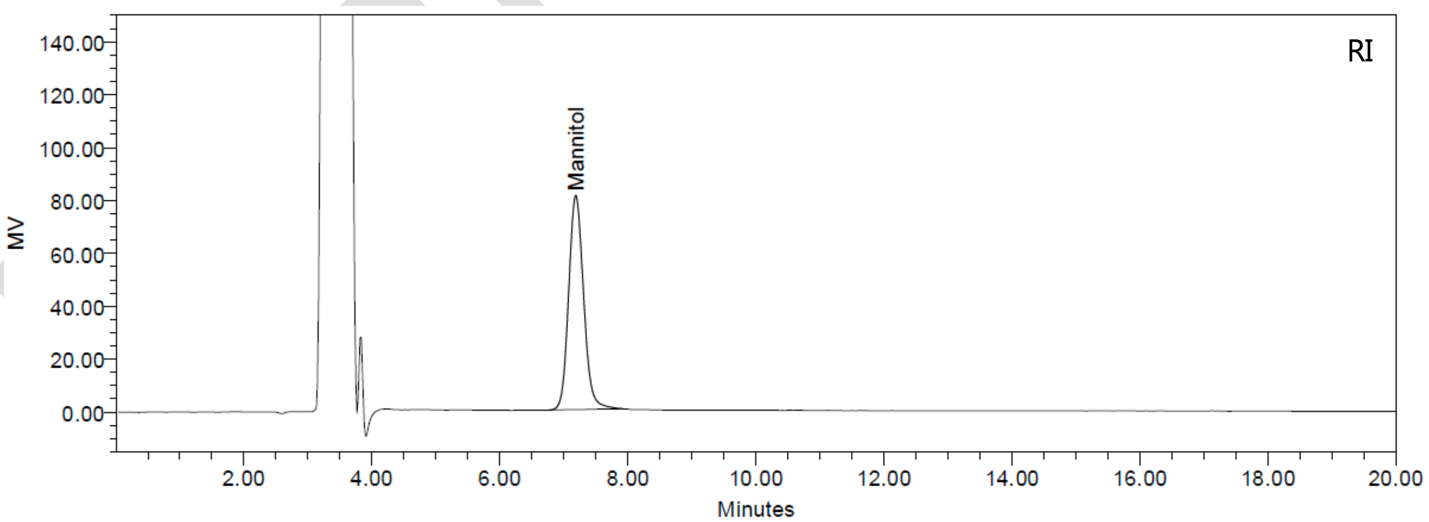


**Supplementary Figure 1**. Chromatogram profiles of adenosine (A), cordycepin (B) and mannitol (C) standards.


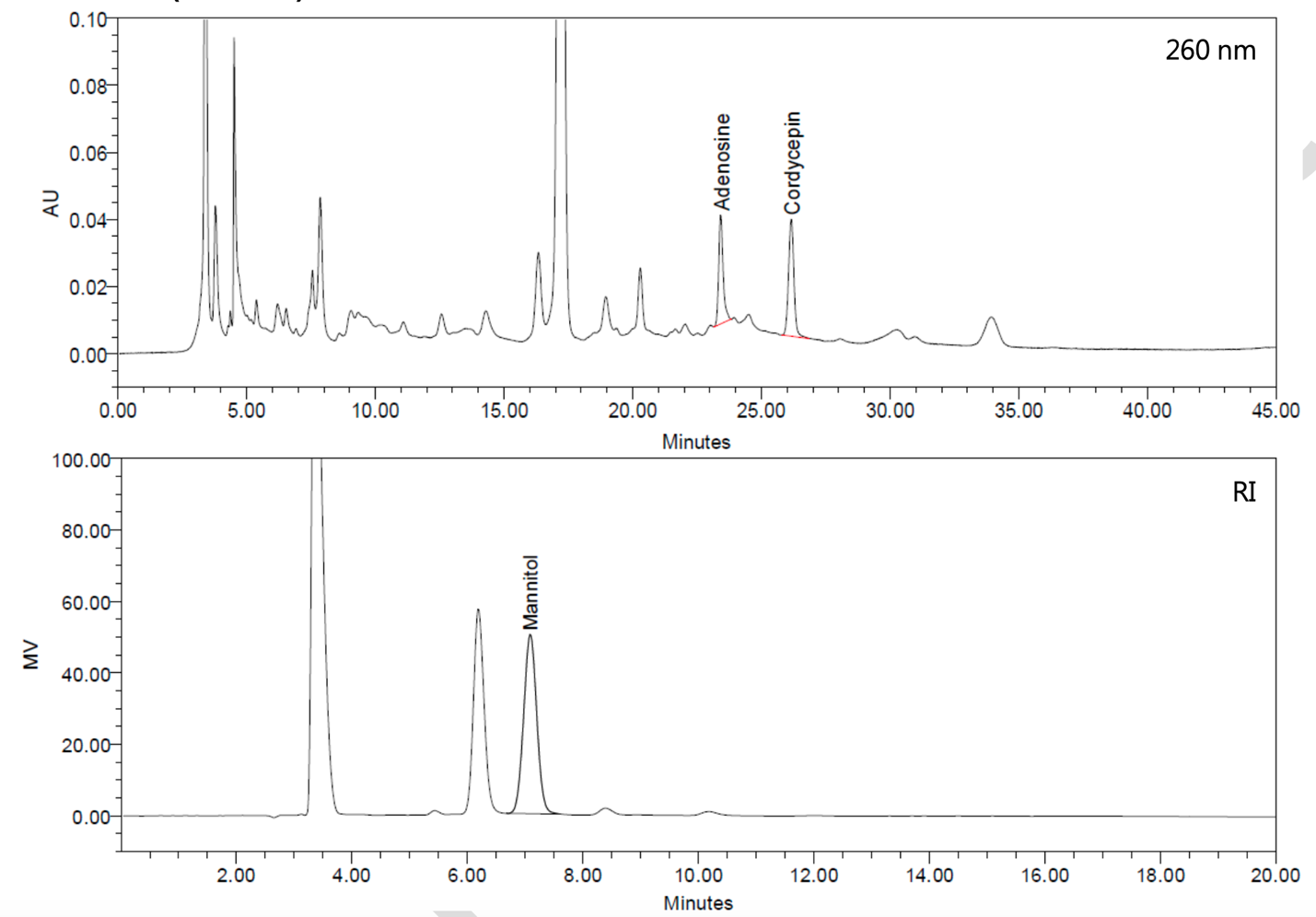


**Supplementary Figure 2**. Chromatogram profiles of the CM-AM formula.

A


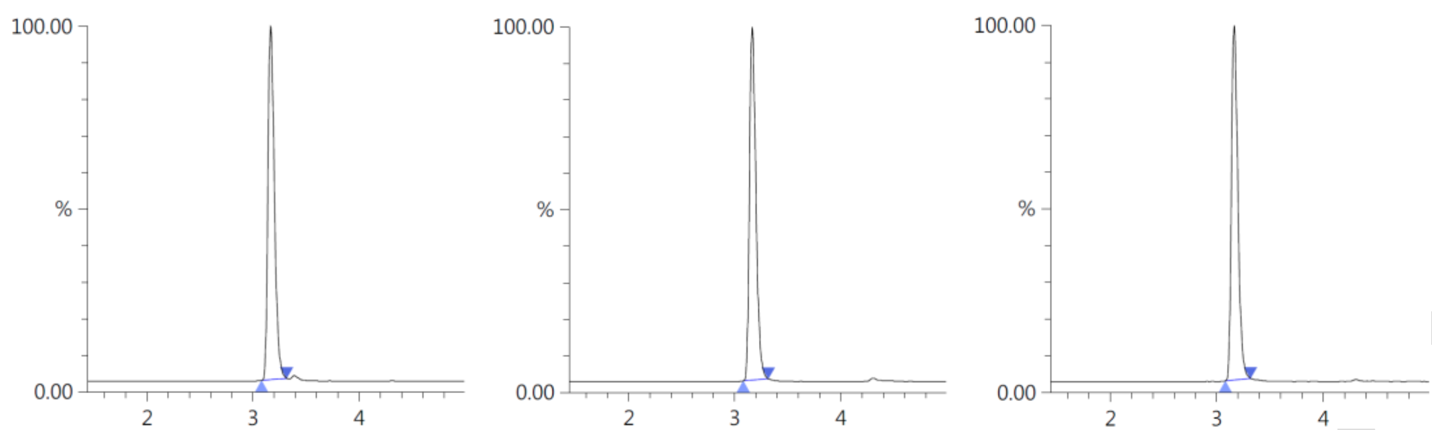


*m/z* 234.90 > 216.95 *m/z* 234.90 > 151.00 *m/z* 234.90 > 161.05

B


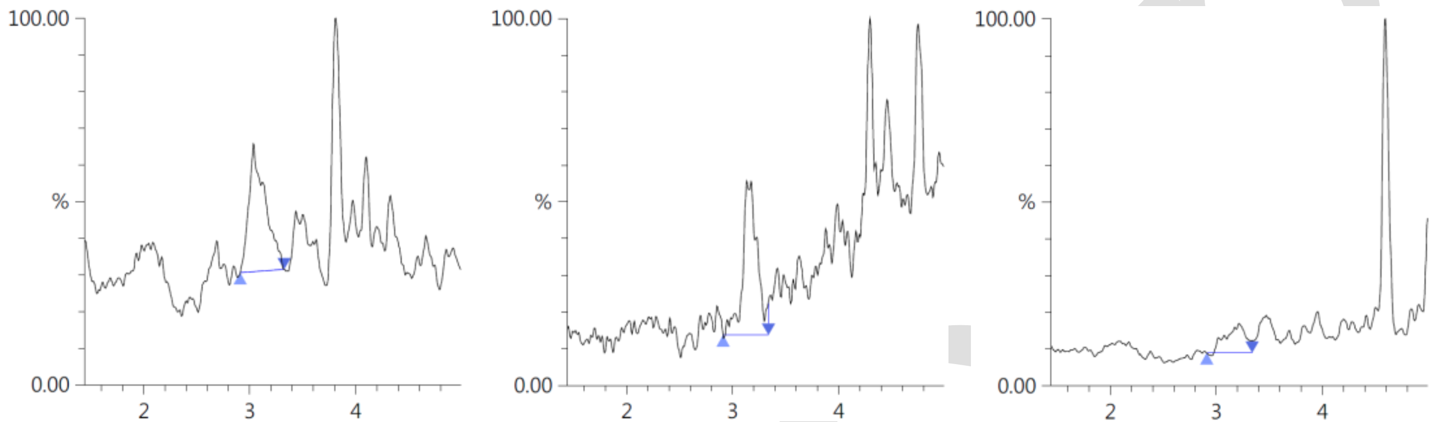


*m/z* 234.90 > 216.95 *m/z* 234.90 > 151.00 *m/z* 234.90 > 161.05

**Supplementary Figure 3**. The LC/MS/MS profiles of armillarisin A standard (A) and the CM-AM formula (B). The concentration of armillarisin A in the CM-AM formula was 0.07 ppb.
